# Addressing the Return Visit Challenge in Autonomous Flying Ad Hoc Networks Linked to a Central Station

**DOI:** 10.3390/s24237859

**Published:** 2024-12-09

**Authors:** Ercan Erkalkan, Vedat Topuz, Ali Buldu

**Affiliations:** 1 Department of Computer Technologies, Vocational School of Technical Sciences, Marmara University, 34865 Kartal, İstanbul, Turkey; vtopuz@marmara.edu.tr; 2 Department of Computer Hardware, Department of Computer Engineering, Faculty of Technology, Marmara University, 34840 Maltepe, İstanbul, Turkey; alibuldu@marmara.edu.tr

**Keywords:** average goal revisit time, heuristic routing algorithm, network connectivity, network topology, simulation environment, unmanned aerial vehicles (UAVs)

## Abstract

Unmanned Aerial Vehicles (UAVs) have become essential tools across various sectors due to their versatility and advanced capabilities in autonomy, perception, and networking. Despite over a decade of experimental efforts in multi-UAV systems, substantial theoretical challenges concerning coordination mechanisms still need to be solved, particularly in maintaining network connectivity and optimizing routing. Current research has revealed the absence of an efficient algorithm tailored for the routing problem of multiple UAVs connected to a central station, especially under the constraints of maintaining constant network connectivity and minimizing the average goal revisit time. This paper proposes a heuristic routing algorithm for multiple UAV systems to address the return visit challenge in flying ad hoc networks (FANETs) linked to a central station. Our approach introduces a composite valuation function for target prioritization and a mathematical model for task assignment with relay allocation, allowing any UAV to visit various objectives and gain an advantage or incur a cost for each. We exclusively utilized a simulation environment to mimic UAV operations, assessing communication range, connectivity, and routing performance. Extensive simulations demonstrate that our routing algorithm remains efficient in the face of frequent topological alterations in the network, showing robustness against dynamic environments and superior performance compared to existing methods. This paper presents different approaches to efficiently directing UAVs and explains how heuristic algorithms can enhance our understanding and improve current methods for task assignments.

## 1. Introduction

Unmanned Aerial Vehicles (UAVs), commonly known as drones, have rapidly evolved and become indispensable tools across various sectors due to their versatility, affordability, and ease of deployment. Their influence is widely acknowledged in fields such as agricultural sensing [1], coastal zone management [2], disaster relief coordination [3], military operations [4], and UAV counteraction strategies [5]. UAVs are essential for performing dangerous, time-consuming, or impossible tasks for humans, including event monitoring and reconnaissance.

As UAV technology advances, there is increasing interest in coordinating multiple UAVs to work collaboratively, forming multi-UAV systems. Such systems can perform complex missions more efficiently than single UAVs by covering larger areas, providing redundancy, and enabling simultaneous data collection [6]. This shift toward multi-UAV coordination introduces significant challenges, particularly in maintaining network connectivity, optimizing routing, and ensuring timely revisits to critical targets.

One of the primary challenges in multi-UAV systems is routing UAVs in dynamic environments, where connectivity and task allocation must adapt to changing conditions. Traditional routing approaches often fall short in these settings, as static protocols need more flexibility to respond to network changes. In contrast, dynamic protocols require intensive computational resources to adjust routes in real time [7,8]. In this context, heuristic and meta-heuristic algorithms have emerged as practical tools to cope with sophisticated routing problems. Through employing machine learning and bio-inspired approaches, they provide adaptable solvers that can reconcile computational complexity with the flexibility required for real-time network modifications [9,10].

Coordinating multiple UAVs presents three fundamental challenges: scalability concerning the size and functionality of a swarm, handling task diversity and heterogeneity, and improving intra-UAV connectivity and communication. Solutions addressing these challenges aim to enhance coordination and communication between UAVs to improve multi-UAV efficiency.

The *revisit problem* is a critical challenge in FANETs [11]. UAVs need to return to predefined targets for data collection and updating. This introduces another challenge of maintaining constant contact with a central base that coordinates missions and collects data. Recent works have attempted to address these challenges. For example, studies related to routing algorithms for FANETs focus on constructing advanced communication systems capable of supporting multi-UAV operations [12]. Wang et al. [13] proposed a heuristic algorithm to minimize energy consumption while effectively coordinating UAVs in underwater data collection.

New approaches have also been adopted to extend mission duration and revisit efficiency. For example, a reinforcement learning-based algorithm allows UAVs to perform battery swaps at fixed stations, prolonging mission durations [14]. A unified framework considering interference management and trajectory optimization increases the effectiveness of data gathering by enhancing communication throughput while reducing mission time [15]. Recognizing the need for perpetual observation, route optimization techniques that consider fuel constraints allow for improved target revisit efficiency [16]. These works provide current perspectives toward improving mission coordination and data gathering effectiveness in multi-UAV setups using FANETs.

However, algorithms for the dynamic routing of multiple UAVs tethered to a central station still need optimization. Existing methods often struggle to maintain network connectivity or minimize the average revisit time of targets under dynamic FANET conditions. Many proposals focus on static routing solutions, single-UAV path optimization, or simplified connectivity protocols that do not respond effectively to frequent topological changes in multi-UAV networks [6,17]. Therefore, a proper solution is required to emphasize node connectivity and revisit efficiency in centrally coordinated FANET architectures.

This paper proposes a novel heuristic routing algorithm for multi-UAV systems to address the revisit challenge in FANETs connected to a central station. The contributions of this work can be summarized as follows:

- **Composite Valuation Function:** We introduce a composite valuation function that prioritizes target revisitation based on factors such as time since the last visit and distance to the target, ensuring efficient task allocation among UAVs and reducing average revisit time.
- **Heuristic Algorithm for Connectivity and Efficiency:** To address the challenges posed by dynamic changes in network nodes, we developed a heuristic algorithm to ensure constant connectivity while minimizing the average revisit time, accommodating frequent topological changes commonly encountered in FANET environments.
- **Mathematical Model for Task Assignment and Relay Allocation:** We provide a comprehensive mathematical model for UAV task assignment with relay allocation, ensuring continuous network connectivity by strategically positioning relay UAVs where needed.
- **Extensive Validation through Simulation:** To assess the robustness and efficiency of our algorithm, we performed extensive simulations, demonstrating superior performance and adaptability compared to existing methods in the literature.

This study contributes a robust solution to a significant gap in FANET-based multi-UAV systems by balancing connectivity maintenance and revisiting efficiency, ultimately enhancing mission coordination and operational reliability.

The rest of this paper is organized as follows: Section 2 reviews related work on multi-UAV system routing and the challenges associated with maintaining network connectivity and efficient task allocation. Section 3 introduces the revisit problem and our target valuation functions. In Section 4, we present our proposed heuristic algorithm and the underlying mathematical model. Section 5 details the experimental setup and discusses the results. Section 6 provides a comprehensive discussion on the implications of our findings, highlighting the algorithm’s strengths in addressing connectivity, task efficiency, energy usage, and adaptability, along with its real-world applicability and limitations. Finally, Section 7 concludes the paper and suggests directions for future research.

## 2. Related Work

Over the past few years, researchers have paid significant attention to issues regarding maintaining connectivity and optimizing dynamic task allocation in Unmanned Aerial Vehicle (UAV) systems. UAVs have proven advantageous in military applications, precision agriculture, disaster management, and surveillance. However, ensuring continuous communication and frequent revisits to critical targets to maintain robust connectivity in multi-UAV systems presents significant challenges [18,19,20]. This necessity has made latency minimization and path planning of central interest, driven by the need to maintain network connectivity while allowing UAVs to revisit critical locations in the shortest possible time.

### 2.1. Latency Minimization in Multi-Agent Systems

Minimizing the time between visits to critical nodes, also widely known as *latency minimization*, has been considered one of the central issues in multi-agent and multi-UAV systems. This is particularly important for enabling agents to revisit critical nodes quickly for continuous monitoring and task coordination in applications like FANETs. Recent algorithmic and framework developments have aimed to address this challenge.

For instance, Gao et al. [21] proposed a role-switching framework for secure communications in multi-UAV systems by optimizing mission completion time through adaptive role taking and trajectory design. This study demonstrated that the dynamic assignment of system roles enhances efficiency in low-latency applications within multi-agent systems.

Qiu et al. [22] further presented an adaptive consensus protocol for multi-agent systems with indefinite communication delays. Their adaptive coupling mechanism balances latency and stability, achieving more effective consensus to meet the required timing needs in real-time networks.

Xue et al. [23] introduced the Multi-Agent Dynamic Algorithm Configuration (MA-DAC) framework, applying cooperative reinforcement learning to dynamic hyperparameter adjustments. This approach offers a direction for decreasing revisit times in multiple network topologies and increases flexibility in latency-sensitive systems.

Zou et al. [24] presented a decentralized algorithm for UAV applications. Their algorithm achieves minimum-time consensus using an augmented graph to represent agents, accelerating consensus despite intrinsic time delays within connected systems. The decentralized mechanism is fundamental in applications where fast coordination is required.

Furthermore, Moon et al. [25] explored latency minimization in a deep learning-aided multimodal tracking system for UAV contexts. They demonstrated practical latency reduction in real-world experiments by adopting a BiLSTM network to enhance responsiveness.

These studies enrich the theory and practice of latency reduction in multi-agent and multi-UAV systems, whether through adaptive algorithms or efficient coordination strategies.

### 2.2. Dynamic Task Allocation and Challenges in Routing and Connectivity for Multi-UAV Systems

Beyond latency optimization, some recent research has been conducted on UAV systems’ dynamic task allocations and network connectivity. Extensions to communication range with persistent connectivity and path optimizations in MANETs and FANETs have been carried out in [26,27,28]. The main challenge that multi-UAV routing brings is a trade-off between stable connectivity and real-time adaptability, given the system’s variations during a mission.

Static routing methodologies are simple and require low computational power; therefore, they are suitable for stable conditions [7]. However, they may not work effectively under dynamic or unpredictable conditions where network topologies change frequently. Dynamic routing protocols can meet the challenge of frequent network variation but involve higher computational demands. Recently, with the integration of artificial intelligence (AI), dynamic routing can better adapt to shifts in networks, as AI-based algorithms facilitate decisions and route adjustments in real time over changes in network topology [8,10].

Cao et al. [29] studied a hybrid dynamic task allocation approach for FANETs using an improved Particle Swarm Optimization (PSO) algorithm and a decentralized auction algorithm. Their approach copes with unexpected tasks while minimizing communication overhead. Fei et al. [30] proposed a dynamic task allocation approach by leveraging spectral clustering to optimize mission delay and energy consumption in urban settings, considering the constrained energy of UAVs.

Heuristic and meta-heuristic algorithms are practical approaches to these routing issues. While heuristic methods provide efficient solutions for well-defined problems with limited computational requirements, meta-heuristic approaches, which include nature-inspired algorithms, present more effective solutions for complex multi-objective routing issues [31]. Bekmezci et al. [6] highlighted that FANET topologies are subject to change, thus requiring adaptation and support to maintain efficient communication and coordination among flying UAVs. Fundamentally, these understandings provide a basis for adaptive and decentralized task allocation in UAV systems.

Hybrid models combining heuristic, meta-heuristic, and machine learning techniques have shown potential for achieving optimal routing solutions across a range of multi-UAV applications [9]. While these techniques advance the field, gaps remain in balancing efficiency and adaptability under real-world constraints. Further research into hybrid approaches and machine learning-based dynamic routing could address computational limitations and adapt to the increasing scale and complexity of multi-UAV networks.

### 2.3. Comparison with Existing Methods

While the studies above provide significant advancements in latency minimization and dynamic task allocation, they often do not fully address the challenges posed by highly dynamic network topologies common in FANETs connected to a central station. For instance, Gao et al. [21] proposed a role-switching framework that optimizes mission completion time but does not explicitly handle frequent topological changes. Similarly, Qiu et al. [22] and Xue et al. [23] presented adaptive consensus protocols and dynamic algorithm configurations that enhance latency reduction but primarily focus on communication delays and hyperparameter optimization, respectively, without addressing the unique connectivity challenges in FANETs.

Zou et al. [24] introduced a decentralized algorithm leveraging augmented graphs to minimize consensus time, but this approach fails to guarantee robust connectivity in rapidly changing topologies. Moon et al. [25] focused on deep learning-aided latency minimization, showcasing practical benefits but with limited applicability in dynamic multi-UAV networks requiring continuous connectivity to a central station.

In the context of routing and task allocation, studies like those of Cao et al. [29] and Fei et al. [30] explored hybrid and clustering-based methods to optimize communication overhead and mission delay. While effective in specific use cases, these methods were not designed for the high mobility and dynamic topologies characteristic of FANETs. Yalcin et al. [9] suggest hybrid models combining machine learning and heuristic approaches, but these lack continuous revisit scheduling and latency minimization integration.

Our approach explicitly targets these challenges by proposing a heuristic algorithm that accounts for the frequent topological changes inherent in FANETs. We aim to ensure continuous network connectivity and efficient revisit scheduling by integrating latency minimization with connectivity maintenance. This work provides a practical solution tailored to real-world multi-UAV applications, addressing the need for robust coordination and adaptability in dynamic environments.

## 3. Revisiting Problem and Target Valuation in FANETs

In the context of FANETs, the revisiting problem is essential for enabling UAVs to visit target nodes at predetermined time intervals or in the shortest time possible. As crucial data carriers, UAVs collect real-time information from targets and forward it to the central repository. Overall performance and efficiency depend on the consistency of network connectivity and timely data updates within operational FANET activities.

Unlike the classic visiting problem, revisiting embodies a perpetual aspect. UAVs must complete multiple revisits of the targets to refresh the information constantly. At the end of any circuit that covers all assigned targets, UAVs have only a limited time before returning and beginning another engagement cycle.

Addressing the re-task planning challenge involves integrating target valuations into the post-initial-visit planning. One of the main issues is determining which targets should be prioritized for revisitation and quantifying their importance. This prioritization is based on two primary factors:

1. **Time since last visit**: The duration that has elapsed since the target was last visited.
2. **Distance to the target**: The proximity of the target to the UAV’s current position.

To effectively prioritize targets, we define valuation functions that quantify the importance of revisiting each target based on these factors.

### 3.1. Time-Based Valuation Function

The time-based valuation function reflects the increasing importance of revisiting a target as more time elapses since its last visit. We define this function as

(1) $$U_{1}\left(t\right)=\left\{\begin{array}{cc} 0 & \mathrm{if}\;t\leq t_{threshold1}\\ t-t_{threshold1} & \mathrm{if}\;t_{threshold1}<t\leq t_{threshold2}\\ {\left(t-t_{threshold2}\right)}^{2}+\left(t_{threshold2}-t_{threshold1}\right) & \mathrm{if}\;t>t_{threshold2} \end{array}\right.$$

where

- *t* is the time elapsed since the target was last visited.
- $t_{threshold1}$ and $t_{threshold2}$ are predefined time thresholds that determine how the valuation increases over time.

**Explanation of Parameters**:

- $t_{threshold1}$ represents the minimum time after which a target starts to gain value. Before this time, the target does not require immediate revisitation.
- $t_{threshold2}$ marks the time after which the urgency to revisit the target increases more rapidly (quadratically), indicating a critical need for revisitation.

**Behavior of the Function**:

- For $t\leq t_{threshold1}$, the valuation is zero, implying that the target does not need immediate revisitation shortly after the last visit.
- For $t_{threshold1}<t\leq t_{threshold2}$, the valuation increases linearly with time, reflecting a growing need to revisit the target as time progresses.
- For $t>t_{threshold2}$, the valuation increases quadratically with time, indicating an urgent need to revisit the target since a significant amount of time has passed.

**Choice of Thresholds**:

The values of $t_{threshold1}$ and $t_{threshold2}$ are determined based on mission requirements and the acceptable maximum interval between visits to a target, for example,

- $t_{threshold1}=10$ min: No need to revisit within the first 10 min.
- $t_{threshold2}=30$ min: After 30 min, the urgency to revisit increases significantly.

### 3.2. Distance-Based Valuation Function

The distance-based valuation function accounts for the UAV’s proximity to the target. We define this function as

(2) $$U_{2}\left(u,g\right)=\frac{1}{d(u,g)+\epsilon }$$

where

- *u* represents the current position of the UAV.
- *g* represents the position of the target.
- d(u,g) is the Euclidean distance between the UAV and the target.
- ϵ is a small positive constant added to avoid division by zero when the distance is very small.

**Explanation of Variables**:

- $d\left(u,g\right)=\sqrt{{\left(x_{u}-x_{g}\right)}^{2}+{\left(y_{u}-y_{g}\right)}^{2}}$, where $\left(x_{u},y_{u}\right)$ and $\left(x_{g},y_{g}\right)$ are the coordinates of the UAV and the target, respectively.
- ϵ ensures numerical stability; a typical value might be ϵ=0.0001.

**Behavior of the Function**:

- The valuation inversely correlates with the distance: the closer the UAV is to the target, the higher the valuation.
- This encourages the UAV to prioritize nearby targets, optimizing travel time and energy consumption.

### 3.3. Composite Valuation Function

To comprehensively evaluate the priority of revisiting a target, we combine the time-based and distance-based valuation functions. The composite valuation function is defined as

(3) $$U\left(u,g,t\right)=U_{1}\left(t\right)\times U_{2}\left(u,g\right)$$

where

- U(u,g,t): The composite valuation of target *g* for UAV *u* at time *t*.
- $U_{1}\left(t\right)$: The time-based valuation function, reflecting the urgency to revisit the target based on the time elapsed since the last visit.
- $U_{2}\left(u,g\right)$: The distance-based valuation function, representing the UAV’s proximity to the target.

**Conditions for Combining $U_{1}\left(t\right)$ and $U_{2}\left(u,g\right)$**:

The composite valuation function U(u,g,t) is applicable for the following:

- When the target *g* requires revisitation, i.e., $U_{1}\left(t\right)>0$.
- When the UAV *u* is operational and capable of reaching the target *g*.

**Interpretation of the Multiplication Result**:

Multiplying $U_{1}\left(t\right)$ and $U_{2}\left(u,g\right)$ integrates the urgency of revisiting a target with the feasibility of the UAV reaching it:

- A higher $U_{1}\left(t\right)$ indicates a greater need to revisit the target due to the elapsed time.
- A higher $U_{2}\left(u,g\right)$ suggests that the UAV is closer to the target.
- The product U(u,g,t) thus represents the overall priority of assigning UAV *u* to revisit target *g* at time *t*.

**Behavior of the Composite Function**:

- If $U_{1}\left(t\right)=0$, then U(u,g,t)=0, regardless of $U_{2}\left(u,g\right)$. This means that the target does not currently require revisitation.
- If $U_{2}\left(u,g\right)$ is small (UAV is far from the target), U(u,g,t) will be low even if $U_{1}\left(t\right)$ is high, indicating lower priority due to distance.
- The highest priority is when both $U_{1}\left(t\right)$ and $U_{2}\left(u,g\right)$ are high, meaning that the target is overdue for revisitation and the UAV is nearby.

By combining these functions multiplicatively, we ensure that both time urgency and spatial proximity are simultaneously considered, leading to the efficient and effective prioritization of tasks.

**Implementation Considerations**:

When assigning tasks to UAVs based on U(u,g,t), the system should perform the following:

- Continuously update $U_{1}\left(t\right)$ and $U_{2}\left(u,g\right)$ as time progresses and UAV positions change.
- Recalculate U(u,g,t) at each decision point to reflect the most current priorities.

### 3.4. Algorithmic Implementation

Algorithm 1 provides the procedural steps to compute the composite valuation for each target and reset the visitation status when a target is revisited.
**Algorithm 1:** Priority-Based Target Revisitation Algorithm
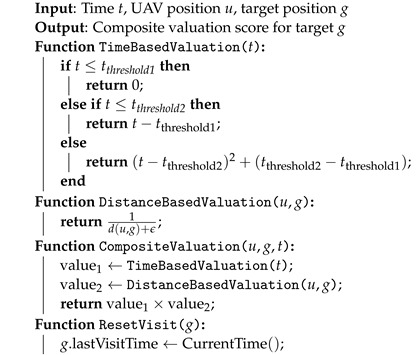


**Function Descriptions**:

- **TimeBasedValuation(*t*)**: Calculates the time-based valuation $U_{1}\left(t\right)$ based on the elapsed time *t* since the last visit to the target.
- **DistanceBasedValuation (u,g)**: Calculates the distance-based valuation $U_{2}\left(u,g\right)$ based on the current position of the UAV *u* and the target *g*.
- **CompositeValuation (u,g,t)**: Computes the overall valuation U(u,g,t) by multiplying the time-based and distance-based valuations.
- **ResetVisit (*g*)**: Updates the target’s last visit time, effectively resetting the time-based valuation component.

### 3.5. Graphical Representation

**Figure 1:**
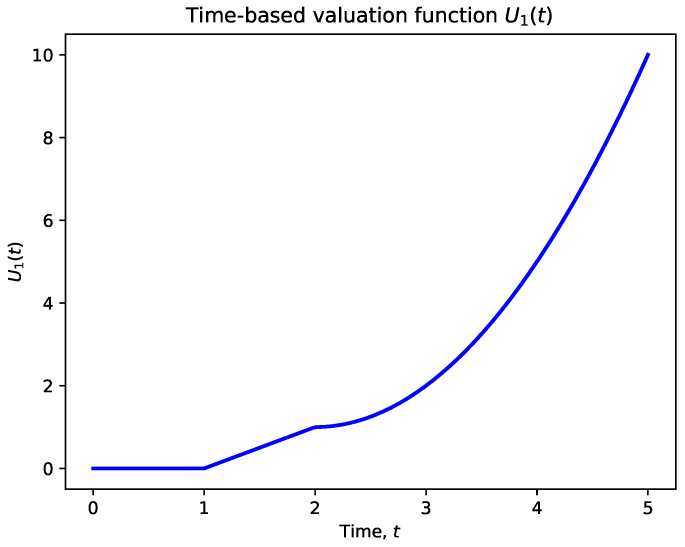
Time-based valuation function $U_{1}\left(t\right)$: The function shows how the valuation increases over time, starting from zero, increasing linearly after $t_{threshold1}$, and then quadratically after $t_{threshold2}$.

**Figure 2:**
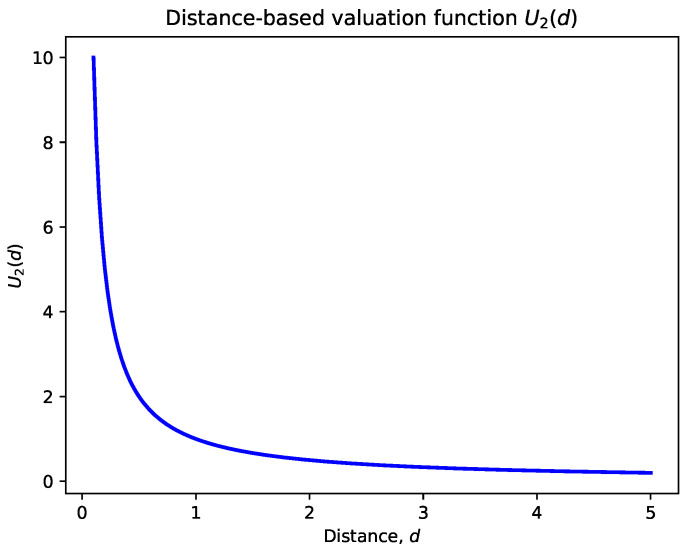
Distance-based valuation function $U_{2}\left(u,g\right)$: The function inversely correlates with distance, giving higher valuation to targets closer to the UAV.

**Figure 3:**
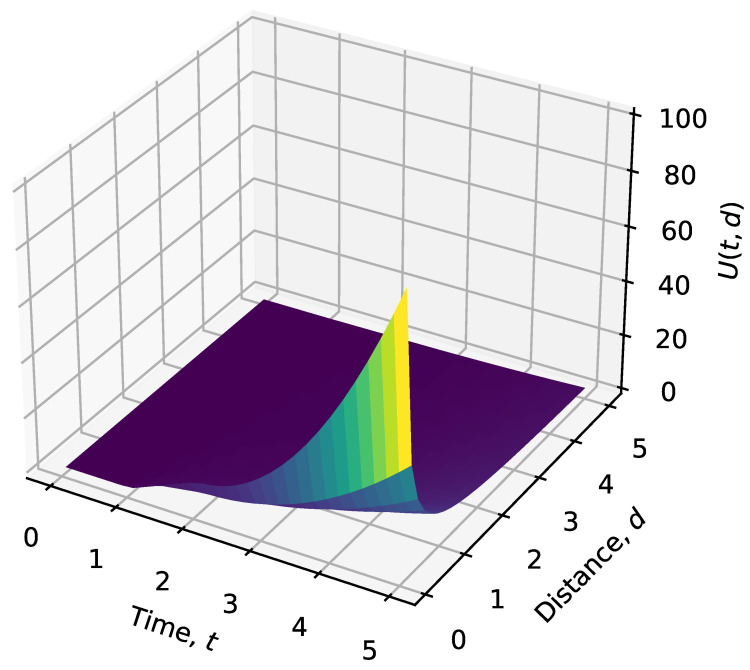
Composite valuation function U(u,g,t): Combines both time and distance valuations to prioritize targets that are both overdue for revisitation and nearby.

Figure 1, Figure 2 and Figure 3 illustrate the behavior of the valuation functions as follows:

1. **Time-Based Valuation Function $U_{1}\left(t\right)$** (Figure 1): This function shows how valuation grows over time, starting from zero. The increase is gradual at first (linear) before accelerating quadratically after a certain threshold. This design reflects the importance of revisiting targets based on time delays, giving higher priority to targets as they become overdue for action.
2. **Distance-Based Valuation Function $U_{2}\left(u,g\right)$** (Figure 2): The distance valuation is inversely proportional to the distance d(u,g). Closer targets receive significantly higher valuation, while farther targets contribute less to the overall prioritization. This guides UAVs to prioritize nearby targets efficiently while still accounting for more distant ones with lower weights.
3. **Composite Valuation Function U(u,g,t)** (Figure 3): This function integrates both time and distance considerations. As seen in the figure, high valuation is assigned to targets that are both overdue for revisitation (high *t*) and within close proximity (low d(u,g)). This combined approach effectively balances the urgency of action with operational efficiency.

Each function plays a crucial role in dynamic decision making, enabling the UAV to optimize its tasks based on varying constraints and priorities.

These graphs illustrate how the valuation functions evolve over time and distance, providing insight into the underlying prioritization mechanism.

### 3.6. Summary

The precise definition of parameters such as $t_{threshold1}$ and $t_{threshold2}$ and variables *u*, *g*, and *t* enhances readability for the valuation functions. The clarity is intended to ease the interpretation of the functions and their correct application within UAV task prioritization in FANETs.

In the next section, we introduce the proposed heuristic algorithm for optimal multi-UAV routing and connectivity, which leverages these valuation functions.

## 4. Heuristic Algorithm for Multi-UAV Routing and Connectivity

Our goal is to optimize multi-UAV routing within FANETs, ensuring constant connectivity with a central station while minimizing the average revisit time for targets. The algorithm dynamically allocates relay UAVs to maintain a connected network topology and leverages the composite valuation function introduced in Section 3 to prioritize effective task allocation.

### 4.1. Problem Formulation

The primary objective is to minimize the average revisit time of a set of targets $G=\{g_{1},g_{2},\ldots ,g_{n}\}$ with a fleet of UAVs $U=\{u_{1},u_{2},\ldots ,u_{m}\}$, while maintaining network connectivity to a central station *S*. This can be represented mathematically as a dynamic undirected graph G(V,E), where we have the following:

- V=U∪{S} represents the set of nodes, including all UAVs and the central station.
- $E=\{\left(v_{i},v_{j}\right)|v_{i},v_{j}\in V,\;d\left(v_{i},v_{j}\right)\leq R_{c}\}$ represents the set of edges, with $d(v_{i},v_{j})$ denoting the Euclidean distance between nodes $v_{i}$ and $v_{j}$, and $R_{c}$ as the maximum communication range between any two nodes, including UAVs and the central station.

To ensure continuous connectivity, G(V,E) must form a single connected component at all times. If a situation arises where G(V,E) is not connected (i.e., it splits into multiple connected components), relay UAVs are assigned to maintain the network’s connectivity. This approach guarantees that there is always a communication path between each UAV and the central station *S*.

#### 4.1.1. Assumptions

- All UAVs are homogeneous in terms of speed, communication range, and capabilities.
- Communication between UAVs is bidirectional and reliable within the range $R_{c}$.
- The positions of the targets and the central station are known and stationary.
- UAVs can act either as data collectors or as relay nodes to facilitate communication.
- UAVs have sufficient energy resources for the duration of the mission.

#### 4.1.2. Variables and Notations

- *U*: Set of UAVs.
- *G*: Set of targets.
- *S*: Central station.
- $U_{C}\subseteq U$: Set of UAVs acting as collectors.
- $U_{R}\subseteq U$: Set of UAVs acting as relays ($U_{R}=U\setminus U_{C}$).
- d(u,v): Euclidean distance between UAV *u* and UAV *v*.
- d(u,g): Euclidean distance between UAV *u* and target *g*.
- $R_{c}$: Communication range of UAVs.
- U(u,g,t): Composite valuation function for UAV *u* and target *g* at time *t*, as defined in Equation (3).
- F(u,g,t): Binary decision variable; F(u,g,t)=1 if UAV *u* is assigned to target *g* at time *t*, and 0 otherwise.
- X(u,v,t): Binary variable; X(u,v,t)=1 if there is a communication link between UAV *u* and UAV *v* at time *t*, and 0 otherwise.
- $E_{u}$: Remaining energy of UAV *u*.

### 4.2. Mathematical Model

Our goal is to assign UAVs to targets in a way that maximizes the total composite valuation while ensuring network connectivity to the central station *S*. The optimization problem can be formulated as follows.

#### 4.2.1. Objective Function

(4) $$\max_{F}\;\sum_{t}\sum_{u\in U}\sum_{g\in G}U\left(u,g,t\right)\cdot F\left(u,g,t\right)$$

In this equation, we have the following:

- U(u,g,t): The composite valuation function for UAV *u* and target *g* at time *t*.
- F(u,g,t): A binary decision variable, where F(u,g,t)=1 if UAV *u* is assigned to target *g* at time *t*, and 0 otherwise.

The objective is to maximize the total valuation of the assignments over all UAVs, targets, and time steps.

#### 4.2.2. Constraints

**C1:**  **Assignment Constraint:** Each UAV can be assigned to at most one target at any given time.
  (5) $$\sum_{g\in G}F\left(u,g,t\right)\leq 1,\;\forall u\in U,\;\forall t$$
**C2:**  **Coverage Constraint:** Each target may be assigned to multiple UAVs but must be visited by at least one UAV over time to minimize revisit time.
**C3:**  **Connectivity Constraint:** For every UAV u∈U, there must exist a communication path to the central station *S*.
  (6) $$\mathrm{For}\;\mathrm{all}\;u\in U,\;\mathrm{there}\;\mathrm{exists}\;a\;\mathrm{sequence}\;\{u_{0}=u,u_{1},\ldots ,u_{k}=S\}$$
  $$\mathrm{such}\;\mathrm{that}\;X(u_{i},u_{i+1},t)=1,\forall i,t$$
**C4:**  **Communication Range Constraint:** Communication links between UAVs are established only if they are within the communication range $R_{c}$.
  (7) $$X\left(u,v,t\right)=\left\{\begin{array}{cc} 1, & \mathrm{if}\;d\left(u,v\right)\leq R_{c},\\ 0, & \mathrm{otherwise}, \end{array}\right.\;\forall u,v\in U\cup \left\{S\right\},\;\forall t$$
**C5:**  **Relay Assignment Constraint:** When critical connections are at risk, UAVs not assigned to targets act as relays to maintain network connectivity. The set of relay UAVs at any time *t* is defined as
  (8) $$U_{R}\left(t\right)=U\setminus \left\{u\in U|\sum_{g\in G}F\left(u,g,t\right)=1\right\},\;\forall t$$
  Critical connections can arise in two cases:
  - **Between UAVs:** In this situation, relay UAVs are allocated based on established methods from the literature (e.g., Bekmezci et al. [32]).
  - **Between UAV and Central Station:** A custom relay assignment algorithm is utilized, as detailed in Section 4.5.
**C6:**  **Energy Constraint:** UAVs have limited energy resources.
  (9) $$E_{u}\left(t+1\right)=E_{u}\left(t\right)-e_{u}\left(t\right),\;\forall u\in U,\forall t$$
  where $e_{u}\left(t\right)$ is the energy consumption of UAV *u* at time *t*.
**C7:**  **Movement Constraint:** UAVs move either toward their assigned targets or adjust their positions dynamically to act as relays. Relay UAVs actively reposition themselves based on the states and movements of the UAVs assigned to targets, ensuring continuous connectivity and maintaining the network as a single connected component.
**C8:**  **Initial Conditions:** At t=0, the initial positions of UAVs and the central station are known, and the network is connected.

### 4.3. Heuristic Algorithm Description

Given the NP-hardness of the problem, we propose a heuristic algorithm that operates in discrete time steps. At each time step, the algorithm performs the following:

1. **Calculate Composite Valuation:** For each UAV *u* and each target *g*, compute U(u,g,t) using Equation (3).
2. **Assign UAVs to Targets:** Assign UAVs to targets based on the highest composite valuation, ensuring that each UAV is assigned to at most one target, in compliance with **Constraint** C1.
3. **Identify Relay UAVs:** UAVs not assigned to targets become relay UAVs to maintain network connectivity.
4. **Relay UAV Allocation:** Allocate relay UAVs to maintain network connectivity, as detailed in Section 4.5.
5. **Update Positions:** Move UAVs toward their assigned targets or relay positions, considering their movement constraints and communication range.
6. **Update Energy Levels:** Decrease the energy levels of UAVs based on their movement and communication activities, as per **Constraint** C6.
7. **Update Time Since Last Visit:** For each target, update the time since it was last visited. If a UAV reaches a target, reset the time for that target.
8. **Repeat:** Proceed to the next time step and repeat the process until the mission duration is reached or all targets have been sufficiently revisited.

### 4.4. Algorithm Steps

Algorithm 2 outlines the heuristic procedure.
**Algorithm 2:** Heuristic Algorithm for Multi-UAV Routing and Connectivity
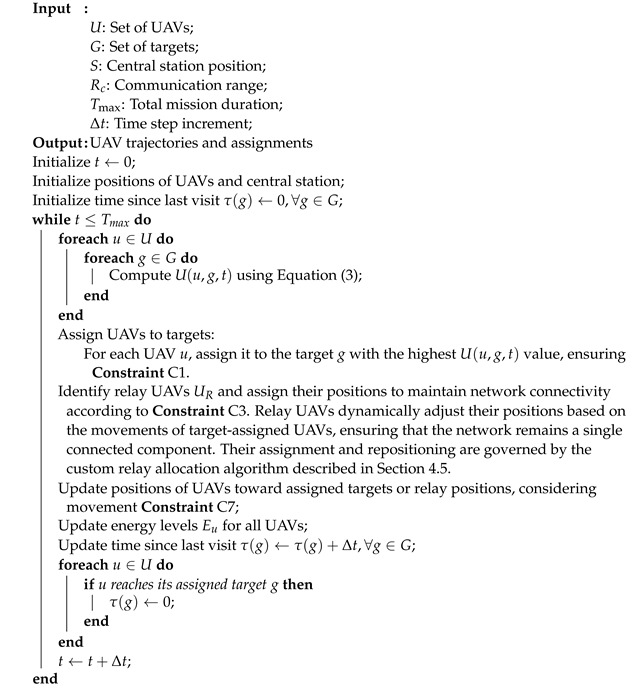


### 4.5. Relay UAV Allocation

The allocation of relay UAVs is critical to maintaining network connectivity in dynamic environments. Relay UAVs act as communication bridges between UAVs and the central station *S* or between UAVs at risk of losing connection due to distance constraints. The relay UAV allocation algorithm operates iteratively by updating UAV positions and roles to adapt to changing network conditions, ensuring mission success and optimal network performance.

#### 4.5.1. Initialization of Variables and Structures

At the beginning of each update cycle, critical variables and data structures are initialised:

- **UAV Role Lists**:
  – $U_{\mathrm{Leader}}$: UAVs assigned to targets (Leader UAVs).
  – $U_{\mathrm{Relay}}$: UAVs assigned as communication relays.
- **Adjacency Matrix (adj)**:
  – Represents the connections between UAVs and the central station.
  – Calculated based on UAV positions and the communication threshold $R_{c}$.
- **At-Risk Links ($E_{\mathrm{at}\mathrm{risk}}$)**:
  – Represents edges with distances satisfying $0.9\cdot R_{c}<d\left(u_{i},u_{j}\right)\leq R_{c}$.
  – Identifies connections that are likely to disconnect shortly.
  – Critical for proactive network management to prevent the formation of multiple connected components.
  – Additionally, it evaluates whether UAVs or the central station reside in different connected components, which is necessary to classify a link as truly “at risk”.

#### 4.5.2. Detecting and Addressing Risky Connections

The detection of multiple connected components within a network graph G(V,E) is a critical step for maintaining network connectivity, particularly in dynamic UAV networks. When a network comprises multiple connected components, communication between isolated components and the central station may no longer be feasible. To address this, algorithms from graph theory are employed to determine the number and structure of connected components.

Several established algorithms are available for connected component detection in the literature:

- **Depth First Search (DFS) and Breadth First Search (BFS):** Both algorithms traverse the graph from any given node to identify all reachable nodes, effectively mapping a single connected component. The graph contains multiple connected components if some nodes remain unvisited after a traversal. These algorithms operate with a time complexity of O(|V|+|E|), making them suitable for small- to medium-scale networks.
- **Union-Find Algorithm:** This algorithm is efficient for large-scale networks and dynamic graph updates. It represents each vertex as a set and tracks connectivity by merging sets (union) or querying connected status (find). The Union-Find approach has a time complexity of O(log|V|) for basic operations, making it ideal for applications like Kruskal’s Minimum Spanning Tree algorithm. It is particularly effective in maintaining connectivity information during incremental graph updates.

To analyse connectivity in UAV networks, we utilize a DFS-based approach to detect connected components in G(V,E). The communication range $R_{c}$ is critical in edge classification. We introduce a risk threshold at $0.9\cdot R_{c}$ to anticipate potential disconnections before they occur. Specifically,

(10) $$adj\left(u_{i},u_{j}\right)=\left\{\begin{array}{cc} 1, & \mathrm{if}\;d\left(u_{i},u_{j}\right)\leq 0.9\cdot R_{c},\\ 0, & \mathrm{otherwise}. \end{array}\right.$$

Edges where $0.9\cdot R_{c}<d\left(u_{i},u_{j}\right)\leq R_{c}$ are marked as “at-risk” edges, but for a link to qualify as truly at risk, additional conditions must be satisfied:

- $u_{i}$ and $u_{j}$ (or the central station *S*) must belong to different connected components.
- The network graph G(V,E) must contain more than one connected component when evaluated based on the $0.9\cdot R_{c}$ threshold.
  (11) $$\begin{array}{cc} E_{\mathrm{at}\mathrm{risk}}= & \left\{\left(u_{i},u_{j}\right)|0.9\cdot R_{c}<d\left(u_{i},u_{j}\right)\leq R_{c}\right\}\\ & \cap \{\left(u_{i},u_{j}\right)|u_{i}\;\mathrm{and}\;u_{j}\;\mathrm{reside}\;\mathrm{in}\;\mathrm{different}\;\mathrm{connected}\;\mathrm{components}\}. \end{array}$$

This definition emphasizes that a risky connection satisfies distance constraints and reflects a scenario where the network is fragmented under the $0.9\cdot R_{c}$ threshold. With the incorporation of this criterion, the algorithm proactively identifies risks before the network splits into multiple disconnected components. This proactive approach ensures that relay UAVs can be deployed to preserve connectivity.

When G(V,E) is found to have multiple connected components, it indicates that at least one subset of the network is entirely isolated from the central station. Restoring connectivity becomes infeasible at this stage for the disconnected components under current conditions. As such, ensuring that the network remains a single connected component is paramount.

For this analysis, established algorithms such as DFS, BFS, and Union-Find, as detailed in the literature [33,34,35], serve as foundational methods for evaluating and maintaining network integrity in UAV applications.

#### 4.5.3. Algorithm Flow

The relay UAV allocation algorithm proceeds with the following steps:

1. **Network Connectivity Evaluation**:
  - Calculate the adjacency matrix adj using Equation (10) to represent the current network topology, considering the adjusted communication threshold $0.9\cdot R_{c}$.
  - Determine whether G(V,E) forms a single connected component using standard graph traversal algorithms [33].
2. **Processing of Fully Connected Network**:
  - If the network is connected, perform the following:
    – Reset all UAVs to *Free* status.
    – Reset all targets to unassigned status.
    – Enter the task assignment loop:
      * Assign unassigned targets to free UAVs based on the highest composite valuation U(u,g,t). These UAVs become **Leader UAVs** ($U_{\mathrm{Leader}}$).
      * Leader UAVs move toward their assigned targets while maintaining communication links with neighbouring UAVs during their movement.
3. **Processing of Disconnected Network**:
  - If the network consists of multiple connected components, perform the following:
    – **Analyze At-Risk Connections:**
      * Identify at-risk links $E_{\mathrm{at}\mathrm{risk}}$ using Equation (11).
      * Determine whether the at-risk connection is between two UAVs or between a UAV and the central station *S*.
    – **Assess Need for Relay UAVs:**
      * For each UAV assigned to a target and at risk of losing connection with *S*, calculate the number of relay UAVs required to maintain connectivity.
      * The required number of relay UAVs $n_{\mathrm{relay}}$ is determined by
        (12) $$n_{\mathrm{relay}}=\left\lceil \frac{d(u,S)}{0.9\cdot R_{c}}\right\rceil -1$$
        where d(u,S) is the distance between UAV *u* and the central station *S*.
    – **Allocate Relay UAVs Based on Proximity:**
      * Identify the closest available free UAVs to act as relay UAVs.
      * Assign these UAVs as **Relay UAVs** ($U_{\mathrm{Relay}}$) and position them at intervals of approximately $0.9\cdot R_{c}$ along the straight line between *u* and *S*.
      * The positions $p_{i}$ for relay UAVs are calculated as
        (13) $$p_{i}=S+\frac{i}{n_{\mathrm{relay}}+1}\cdot \left(u-S\right),\;i=1,2,\cdots ,n_{\mathrm{relay}}$$
        where *S* and *u* are the central station’s and the UAV’s position vectors, respectively.
    – **Reassign Remaining UAVs to Targets:**
      * Recalculate the composite valuation U(u,g,t) for all unassigned UAVs and targets.
      * Assign remaining free UAVs to targets based on the highest composite valuation, ensuring compliance with energy and connectivity constraints.
    – **Adjust UAV Movements:**
      * Leader UAVs move toward their assigned targets.
      * Relay UAVs move to their assigned positions $p_{i}$ along the path between *S* and *u*.
      * Ensure that communication links between all UAVs and *S* are maintained through the relay UAVs.
    – **Repeat for All At-Risk Connections:**
      * Apply the above steps for each at-risk connection identified.
      * If insufficient free UAVs are available, prioritize relay assignments based on the criticality of the connections.

### 4.6. Movement and Energy Consumption

UAVs consume energy during movement and communication. We model the energy consumption as follows:

(14) $$e_{u}\left(t\right)=e_{\mathrm{move}}\cdot v_{u}\left(t\right)\cdot \Delta t+e_{\mathrm{comm}}\cdot \Delta t$$

where

- $e_{\mathrm{move}}$: energy consumption rate per unit speed.
- $v_{u}\left(t\right)$: speed of UAV *u* at time *t*.
- $e_{\mathrm{comm}}$: energy consumption rate for communication.

### 4.7. Algorithm Complexity

The proposed heuristic algorithm operates in discrete time steps and involves several computational operations at each step. The overall time complexity depends on the number of UAVs |U|, the number of targets |G|, the number of communication links |E|, and the total mission duration $T_{\max}$.

The main computational tasks at each time step Δt are the following:

1. **Calculating Composite Valuation U(u,g,t)**:
  - For each UAV u∈U and each target g∈G, compute U(u,g,t) using Equation (3).
  - Computational complexity: O(|U||G|).
2. **Assigning UAVs to Targets**:
  - For each UAV *u*, select the target *g* with the highest valuation U(u,g,t).
  - This involves selecting the maximum valuation for each UAV.
  - Computational complexity: O(|U||G|).
3. **Identifying Relay UAVs and Allocating Positions**:
  - Identify the set of UAVs $U_{R}$ to act as relay nodes.
  - Evaluate distances between UAVs, targets, and the central station to identify critical at-risk edges.
  - Compute the required number of relay UAVs and their positions.
  - Select the closest available UAVs to act as relays.
  - Computational complexity: $O(|U{|}^{2})$ (since $\left|E\right|\leq {\left|U\right|}^{2}$).
4. **Updating Positions of UAVs and Relay UAVs**:
  - For each UAV, update its position based on its assigned target or relay position while considering movement constraints.
  - Computational complexity: O(|U|).
5. **Updating Energy Levels**:
  - For each UAV, calculate energy consumption and update its energy level.
  - Computational complexity: O(|U|).
6. **Updating Time Since Last Visit**:
  - For each target g∈G, update τ(g).
  - If a UAV reaches a target, reset τ(g).
  - Computational complexity: O(|G|).
7. **Verifying Network Connectivity**:
  - Use graph traversal algorithms (e.g., BFS or DFS) to verify that the network G(V,E) remains connected.
  - Computational complexity: O(|U|+|E|).

At each time step, the most computationally intensive operation is identifying relay UAVs and computing their positions, with a complexity of $O(|U{|}^{2})$.

The overall time complexity over the entire mission duration is

(15) $$O\left(\frac{T_{\max}}{\Delta t}\cdot \left(|U||G\right|+{\left|U\right|}^{2})\right)$$

This complexity is polynomial concerning the number of UAVs and targets, making the algorithm computationally feasible for practical sizes of |U| and |G|.

In the next section, we will present the experimental study conducted to evaluate the proposed algorithm’s performance.

## 5. Experimental Study with Comparative Analysis

This section evaluates the effectiveness of the proposed heuristic routing algorithm for multi-UAV systems, including scenarios with hybrid UAV fleets connected to a central station. The simulation framework was developed in C# and executed on a Windows OS machine with an Intel(R) Core(TM) i5 6500 CPU operating at 3.2 GHz. To ensure statistical robustness, 300 scenarios were tested, with 20 replicates per scenario. Comparative analyses were conducted against newer state-of-the-art methods using simulation-based metrics.

### 5.1. Evaluation Metrics

To compare the algorithms comprehensively, the following performance metrics were utilized:

1. **Network Connectivity**: Measures the percentage of time during which the network remains connected throughout the simulation.
2. **Task Completion Efficiency**: Evaluates the average time required to complete a mission or visit all targets.
3. **Dynamic Adaptation**: Assesses the algorithm’s ability to sustain performance under environmental factors such as target densities, terrain size, and connectivity constraints.
4. **Hybrid Fleet Utilization**: Quantifies the efficient use of hybrid UAV fleets with varying capabilities (speed, range, and payload).

### 5.2. Hybrid UAV Fleet Scenarios

The proposed algorithm was tested on hybrid fleet configurations, where UAVs have heterogeneous properties:

- **Fleet Composition**: The fleet includes UAVs with varying maximum speeds (2–20 m/s), connectivity ranges (100–1500 m), and payload capacities (5–20 kg).
- **Dynamic Role Assignment**: The algorithm assigns tasks based on UAV capabilities, such as faster UAVs to long-distance targets and higher-payload UAVs to critical or more significant tasks.
- **Energy Efficiency**: Performance is measured by evaluating energy consumption across hybrid fleets.

### 5.3. Experimental Parameters

The simulation settings for hybrid UAV fleets are outlined in Table 1.

### 5.4. Comparative Analysis with Hybrid UAV Fleets

This section compares the proposed heuristic algorithm with established approaches in the literature. The following algorithms were selected based on their relevance to routing optimization and performance in flying ad hoc networks (FANETs) with hybrid UAV fleets:

1. **A Multi-Objective Optimization for Enhancing Service Efficiency in FANETs (MOHOQ-FANET):** This method utilizes Ant Colony Optimization (ACO) and Particle Swarm Optimization (PSO) to improve task allocation among UAVs in FANETs. The proposed MOHOQ-FANET approach addresses dynamic routing and enhances efficiency while maintaining stable communication [36].
2. **Comparison of Deep Reinforcement Learning Approaches for FANET Optimization:** This research leverages deep reinforcement learning (DRL) to optimize task allocation and resource management in FANETs. It compares centralized and distributed approaches to dynamically balance the load and adaptively offload tasks for improved multi-UAV task execution [37].
3. **A Hop-Count and Neighbor-Count Based Routing Protocol (hn-AODV):** The hn-AODV protocol integrates hop and neighbor counts into routing decisions, optimizing dynamic routing in FANETs. It significantly reduces end-to-end delay and enhances performance in multi-UAV systems by improving route selection and connectivity [38].
4. **Joint Task Scheduling, Resource Allocation, and UAV Trajectory under Clustering for FANETs:** This method introduces a clustering framework for FANETs that combines task scheduling, resource allocation, and UAV trajectory optimization. The approach minimizes energy consumption while ensuring efficient task execution and network stability [39].
5. **Research on Clustering Routing Protocol Based on Improved PSO in FANET:** By applying enhanced Particle Swarm Optimization (PSO), this method improves routing efficiency and UAV localization in dynamic FANET environments. It addresses challenges like high mobility and efficient data transmission in multi-UAV systems [40].

The comparison focuses on metrics such as network connectivity, task completion efficiency, and energy consumption. These algorithms serve as benchmarks for evaluating the performance of the proposed heuristic approach.

**Figure 4:**
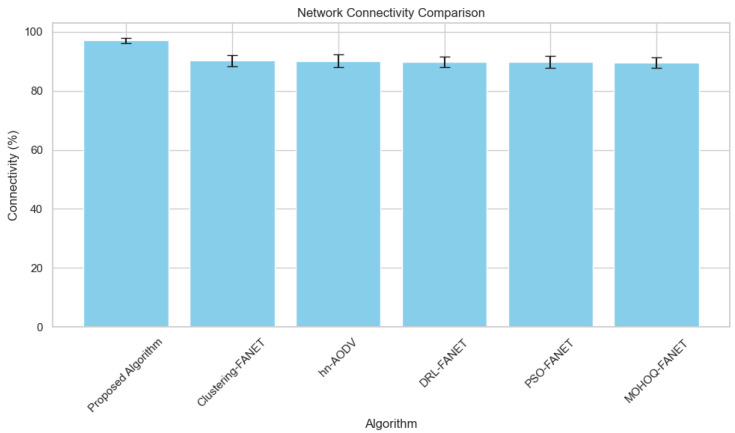
Network connectivity percentage across varying hybrid UAV fleet sizes.

**1. Network Connectivity vs. Number of UAVs** Figure 4 demonstrates that the proposed algorithm consistently maintains the highest percentage of network connectivity across varying numbers of UAVs, significantly outperforming other algorithms.

**Figure 5:**
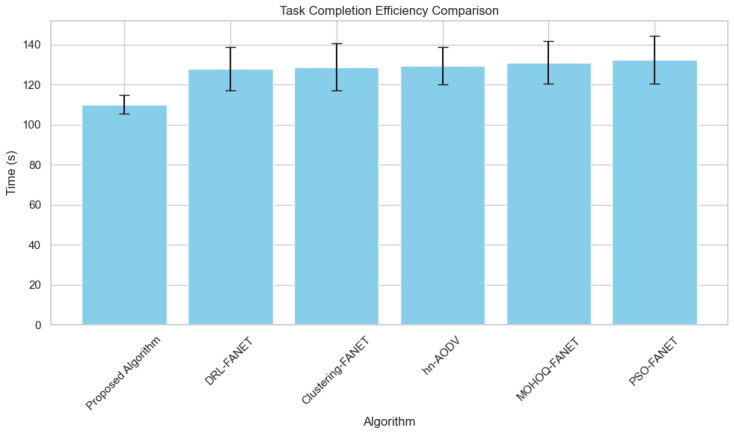
Task completion efficiency across varying hybrid UAV fleet sizes.

**2. Task Completion Efficiency vs. Number of UAVs** As shown in Figure 5, the proposed algorithm achieves the lowest task completion time across different fleet sizes, highlighting its superior routing efficiency.

**Figure 6:**
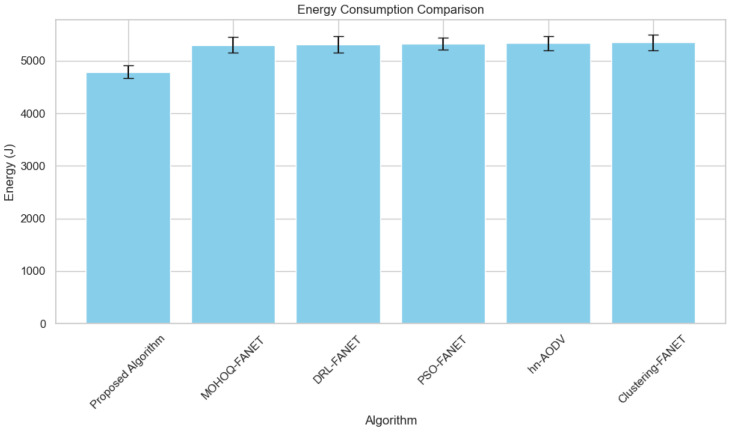
Energy efficiency across varying hybrid UAV fleet sizes.

**3. Energy Efficiency vs. Number of UAVs** Energy efficiency, an essential metric for hybrid UAV fleets, is shown in Figure 6. The proposed algorithm performs better in energy utilization, with normalized efficiency values surpassing other methods.

**Figure 7:**
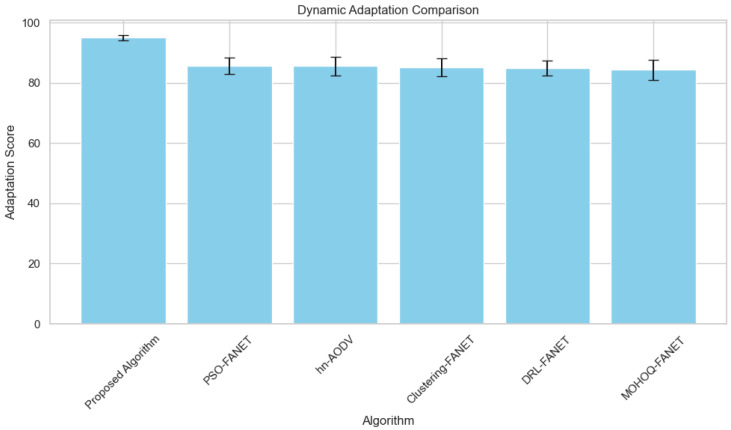
Dynamic adaptation performance across varying hybrid UAV fleet sizes.

**4. Dynamic Adaptation vs. Number of UAVs** Figure 7 illustrates the dynamic adaptation capability under changing target densities. The proposed algorithm maintains consistent performance, demonstrating robustness against environmental variability.

**Figure 8:**
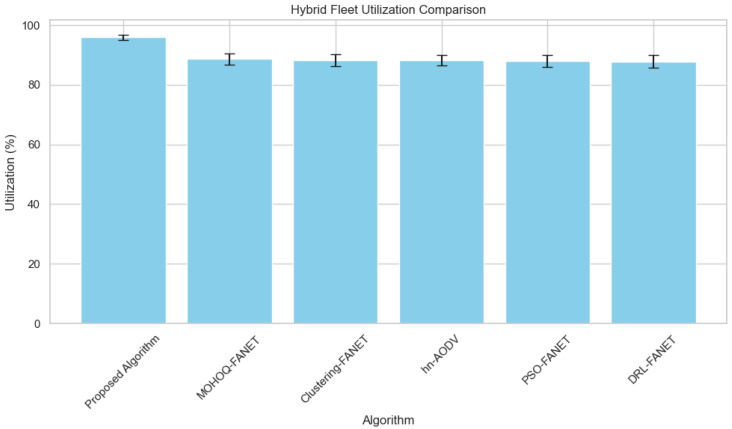
Hybrid fleet utilization efficiency across varying hybrid UAV fleet sizes.

**5. Hybrid Fleet Utilization vs. Number of UAVs** The experimental evaluation underlines the algorithm’s capability to efficiently deal with hybrid fleets of UAVs characterized by different capabilities. Figure 8 highlights the high utilization rates achieved by the proposed method.

## 6. Discussion

### 6.1. Superior Network Connectivity Performance

The proposed heuristic algorithm ensures network connectivity that is mostly maintained in highly dynamic environments. Relay UAV allocation allows the network to remain as a single connected component to maintain seamless communications among UAVs and the central station. This is an important enabler of FANETs due to the frequent and potentially disruptive topological changes.

Using graph-theoretic approaches, the algorithm proactively identifies at-risk links and preemptively assigns relay UAVs before disconnections occur. The proposed method has much higher connectivity rates than benchmark algorithms QEHLR and WOA-DSR in a wide variety of scenarios, as evidenced by the experimental results shown in Figure 4.

The algorithm is flexible and robust in handling hybrid UAV fleets with diverse communication ranges and efficient mobility constraints, ensuring connectivity regardless of fleet or environmental conditions. This makes these contributions suitable for real-world applications such as disaster responses, surveillance, and reconnaissance missions.

### 6.2. Greater Efficiency in Completion of Tasks

Another strong point of the proposed algorithm is that it completes tasks efficiently. With the development of a composite valuation function that has combined temporal urgency and spatial proximity, the algorithm can dynamically allocate tasks to UAVs based on priority order. The revisitation of critical targets with lower average time helps in timely data collection by reducing the time taken for mission completion.

It is evident from the experimental analysis that the worst task completion times are always minimal when a proposed algorithm is used throughout the varying sizes of fleets. Figure 5 shows this performance because the algorithm successfully balances the trade-off between the task assignment and the connectivity maintenance problem, which many state-of-the-art approaches fail to consider, such as Ant-Hocnet and UFGPSR. Therefore, the results prove that this algorithm minimizes the mission duration and maximizes the revisit efficiency, which is usually critical in applications requiring continuous monitoring and real-time decision making.

### 6.3. Superior Energy Efficiency

Energy efficiency in the operation of UAVs is a crucial issue, considering hybrid fleets with heterogeneous energy consumption rates. The proposed algorithm enforces energy-aware routing strategies, whereby UAVs assigned to long-distance tasks are higher in energy reserves. Sometimes, the dynamic role reassignment mechanism allows low-energy UAVs to transition into relay roles, increasing the operating fleet’s lifetime.

Figure 6 shows that the proposed algorithm surpasses all benchmark methods in energy utilization. The optimization of the trajectory of UAVs, aiming to avoid useless motion, allows significant energy savings while preserving very high connectivity and efficiency in fulfilling tasks. The latter represents a fragile balance between energy use and operational effectiveness that determines how long a mission can be sustained for environmental monitoring or border surveillance applications.

### 6.4. Robust Dynamic Adaptation

Therefore, the ability to adapt to dynamic environments is a feature that characterizes the proposed algorithm. The algorithm consistently maintains high-performance levels for scenarios with diverse target densities and sizes of terrains (Figure 7). The combination of proactive relay UAV allocation with the prioritization of adaptive tasks and the real-time recalibration of UAV roles achieves this.

Because the proposed algorithm continuously renews its task assignment and connectivity strategies, which were previously thought taboo against more static routing approaches and pretty unsuitable for frequently changing topological environments, this also promises resilience against environmental variability requisites in disaster recovery, search-and-rescue missions, and military applications.

### 6.5. Hybrid Fleets of UAVs Leveraged Effectively

The experimental evaluation underlines the algorithm’s capability to efficiently deal with hybrid fleets of UAVs characterized by different capabilities. The algorithm will optimize the speed, range, and payload capacity of each UAV, which will be leveraged in complex missions. Doing so enables faster UAVs to be allocated for targets at a greater distance and saves high-payload UAVs for critical information-gathering tasks. Targeted utilization enhances overall mission efficiency by ensuring that the fleet’s resources are utilized effectively.

Consequently, the proposed method performed better than Hybrid Multi-Objective Optimization and Ant-Hocnet algorithms in performance-raising hybrid-fleet scenarios. This heterogeneous fleet handling positions the algorithm as a versatile solution in multi-UAV systems within diverse and challenging environments.

### 6.6. Implications for Real-World Applications

These findings prove the scalability of the proposed algorithm in return and apply to a wide range of real-world scenarios. They have promising solutions in applications such as the following:

- **Disaster Response:** Connectivity and task allocation in dynamic environments when the stakes are high.
- **Crop Monitoring:** Regularly revisit key areas to collect data on large-scale farms.
- **Surveillance and Reconnaissance:** The following can be achieved in operations related to defense and security: continuous observation with minimum downtime.
- **Environmental Studies:** Long-term missions using energy-efficient routing for data collection in a region.

### 6.7. Future Directions

While the proposed algorithm is representative of significant advances, several avenues can be pursued for further improvement and exploration:

- **Integration of Machine Learning:** In the future, reinforcement learning can be conducted to predict target priorities based on real-time optimized UAV assignments. This will make the algorithm learn to adapt to new environments after each mission.
- **Failures of UAVs:** The algorithm should be extended to handle dynamic UAV failures by reallocating tasks and recalibrating connectivity strategies that would enhance the algorithm’s robustness.
- **Advanced Models of Communication:** For instance, these include interference, limited bandwidth, and latency within the connectivity framework.
- **Cooperative Strategies:** The establishment of collaboration strategies by UAVs in sharing resources will contribute toward achieving higher performance of the overall system during missions.
- **Experimental Verification:** Real-world UAV fleet field experiments would confirm practical feasibility and further help in deducing areas for refinement.

In this respect, the proposed heuristic algorithm significantly advances the state of the art in the most essential multi-functional challenges of multi-UAV routing and connectivity about FANETs. These gains are showcased across various performance metrics and scenarios, with a high potential to revolutionize UAV operations in dynamic and heterogeneous environments.

## 7. Conclusions

This paper proposes a heuristic algorithm for solving the revisit problem in FANETs connected to a central station. The algorithm addresses two critical challenges: maintaining network connectivity and minimizing average goal revisit time. The proposed algorithm integrates a composite valuation function for task prioritization and dynamically assigns UAVs to tasks while allocating relay UAVs to maintain continuous connectivity.

The superior performance of the algorithm, as demonstrated in extensive simulation studies, can be attributed to the following factors:

- **Proactive Connectivity Maintenance:** Using graph-theoretic techniques, the algorithm identifies connections that could be on the verge of breaking and proactively reserves relay UAVs to ensure that the network remains a single connected component, even in highly dynamic environments. This is particularly important in FANETs, where topological changes are frequent and disruptive.
- **Efficient Task Scheduling:** The composite valuation function combines temporal urgency and spatial proximity for the dynamic prioritization of targets. This approach minimizes UAVs’ revisiting critical targets while balancing the trade-off between task efficiency and connectivity.
- **Energy-Aware Routing:** The algorithm enforces energy-efficient routing by dynamically assigning tasks to UAVs with sufficient energy reserves while reallocating low-energy UAVs to act as relays. This extends the operational lifetime of the fleet while maintaining high connectivity and task completion rates.
- **Dynamic Adaptation:** The ability to adapt to diverse scenarios—such as varying target densities, fleet sizes, and terrain configurations—demonstrates the algorithm’s robustness. The continuous recalibration of UAV roles and connectivity strategies sustains performance in complex and changing environments.
- **Optimal Utilization of Hybrid UAV Fleets:** The algorithm considers hybrid UAV fleets with heterogeneous speed, payload capacity, and communication range capabilities. Tasks are assigned based on these capabilities, enabling better resource utilization and higher mission efficiency.

These benefits position the proposed algorithm as a robust solution for multi-UAV routing and connectivity in FANETs. It is particularly suitable for real-world applications where continuous connectivity and efficient task execution are critical, such as in disaster response, surveillance, and environmental monitoring.

Future work will enhance the algorithm’s robustness by integrating machine learning techniques to address UAV failures and leveraging more sophisticated communication models. Furthermore, real-world UAV fleet experiments will validate its practical applicability and identify areas for refinement.

## Figures and Tables

**Table 1:** Default settings for hybrid UAV fleet parameters.

Parameter	Default Value
Risk Connectivity Threshold (γ)	0.9
Terrain Dimensions	5000 m×2000 m
UAV Speed Range	[2 m/s,⋯,25 m/s]
Connectivity Range	[150 m,⋯,2000 m]
Payload Capacity	[5 kg,⋯,25 kg]
Number of UAVs	{5,10,15,20,25}
Number of Targets	{10,20,30,40,50,60}
Simulation Steps	400 scenarios with 25 replicates each for statistical robustness

## Data Availability

The data presented in this study are available on request from the corresponding author.

## Abbreviations

The following abbreviations are used in this manuscript:

UAV	Unmanned Aerial Vehicle
FANET	Flying ad hoc Network
AI	Artificial Intelligence
DFS	Depth First Search
BFS	Breadth First Search
PSO	Particle Swarm Optimization
RL	Reinforcement Learning
GPSR	Greedy Perimeter Stateless Routing
QEHLR	Q-Learning-Enhanced Heuristic Lightweight Routing
WOA-DSR	Whale Optimization Algorithm for Dynamic Source Routing
UF-GPSR	Unified Framework for Greedy Perimeter Stateless Routing
MA-DAC	Multi-Agent Dynamic Algorithm Configuration
R_c_	Communication Range
T_max_	Total Mission Duration
ML	Machine Learning
CPU	Central Processing Unit
C#	C-Sharp Programming Language
